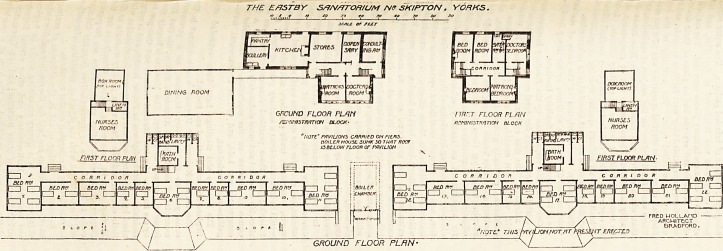# Bradford Union Sanatorium for Pauper Consumptives at Eastby

**Published:** 1904-07-23

**Authors:** 


### BRADFORD UNION SANATORIUM FOR PAUPER CONSUMPTIVES AT EASTBY.

The Bradford guardians are to be congratulated on carrying out their scheme for providing accommodation for their consumptive patients, and we believe this is the first hospital of its kind in England. It is true that private and charitable sanatoria are springing up in all directions, and that in some of these patients are admitted at very low rates of board; but this is an example of what can be done for the very poorest classes by an entirely rate-supported institution; and there cannot be a doubt that other unions will sooner or later follow such a noble example as has been set by Bradford. A plot of land near Eastby was bought for £570. It consists of upwards of seven acres, and is at a high altitude some little distance from the road leading to Barden Moor.

The buildings face south-east, and are sheltered from the north and the west by the formation of the land, and from the east winds by a plantation of firs. These are important points in an English sanatorium, and their consideration shows that the whole matter has received careful attention. The sanatorium consists, or when completed will consist, of two pavilions, between which is the boiler-house, which is sunk so as to be below the floors of the neighbouring blocks. To the north is the administrative block, which contains rooms for the resident medical officer, the matron, and the domestic servants, and to which is attached a large dining-room.

The patients' pavilions are carried on piers, which is the correct method, and each pavilion has in its centre a four-bedded room with a bay having three large windows: on each side of this are two single-bedded rooms, then two double-bedded rooms, and at each end is a five-bedded ward. A corridor runs along the north aspect of the block. Exactly in rear of the centre of the corridor is the block, or rather the projection, containing the closets and the bath-room. The corridor projection is open. The bathroom is lighted from windows in this corridor partition, in addition to windows on the north side, there being a dwarf partition 8 feet high between spray baths and slipper baths. We should like to have seen the whole of this part better cut off from the corridor than it is. Each patient's room has a window to the south opening full width, but these windows do not reach down to the floor level, which we look upon as a fault. The doors are in most cases nearly opposite the windows, and there is an unglazed opening from the room into the corridor. A good deal of cross-ventilation will be thus obtained. Excepting that the central parts of the pavilions are carried up for nurses' rooms, they are of one story only, and the staircases for these nurses' rooms are in the projections already noticed. There are no verandahs. The architect remarks that he does not think them necessary, and that the medical officers of some sanatoria deprecate their use. We are not in agreement with these medical men, as we believe that verandahs are at times, and for some patients, very useful;

THE EASTBY SANATORIUM Nr. SKIPTON, YORKS.

but, like every other part, they must be properly constructed. The dimensions of the rooms are single bedrooms, 120 feet superficial area. The five-bedded rooms contain 85 superficial feet per patient, and the two-bedded rooms 135 feet per patient. Of course much more depends upon the thorough perflation of air than on mere superficial or cubic space; but in the five-bedded rooms we are of opinion that the superficial area is barely enough. Again, whether five, or even two, phthisical patients should occupy the same room may at present be a moot point, but it is one which the future will almost certainly decide in the negative. In saying this we do not forget that the Bradford guardians are pioneers, and that they are spending public money, which may have somewhat hampered them, and that the architect would have to carry out instructions given to him. Nevertheless, when everything has been said from both of these standpoints, it is evident that the administrative department and the various adjuncts would be the same in any case, and that the extra expense in giving every patient a separate room would not have been very great, while the advantage to the patient would have been a real one. Indeed, we may affirm that, where detached huts are impossible, single-bedded wards should be the rule. Each pavilion contains a total of 26 beds.

Electric light will be used throughout the whole of the establishment, the pavilions will be warmed by hot-water coils on the low-pressure system, and the ventilation is by means of open windows.

There is no laundry, as it is intended to have the washing done at the Union Workhouse; in fact, the institution will be carried out as a branch of the Bradford Union Hospital, and no patient will be admitted who has not previously been examined and sent in by the medical officer of the workhouse, though there will be a resident medical officer in the sanatorium. Trained nurses will also be in residence.

The building was formally opened by Sir Francis Powell, Bart. The architect is Mr. Frederick Holland, of Bradford, and the cost was about £10,000.

## Figures and Tables

**Figure f1:**